# Sleep characteristic profiles and the correlation with spectrum of metabolic syndrome among older adult: a cross-sectional study

**DOI:** 10.1186/s12877-022-03074-8

**Published:** 2022-05-11

**Authors:** Xin Liu, Limei Huang, Qiang Wu, Yingwei Chen, Xiuqin Chen, Hao Chen, Junling Gao, Qianyi Xiao

**Affiliations:** 1 Department of Preventive Medicine and Health Education, School of Public Health, Fudan University, 138 Yixueyuan Road, Shanghai, 200032 China; 2 Present Address: Songjiang Center of Disease Prevention and Control, Shanghai, 201620 China; 3 Present Address: Songjiang District Xinqiao Town Community Health Service Center, Shanghai, 201600 China

**Keywords:** Sleep characteristic, Metabolic syndrome, Pittsburgh Sleep Quality Index, Rise time, Older adults

## Abstract

**Background:**

Metabolic Syndrome (MetS) is a common health problem among older adults. Previous studies have revealed the relationship between sleep duration as well as global sleep status and MetS.

**Objectives:**

This study aims to examine the association between the specific sleep characteristic and MetS as well as MetS components among community-dwelling old adults.

**Methods:**

This cross-sectional study included 1499 community residents aged ≥ 60 years. Sleep characteristics were assessed using the Pittsburgh Sleep Quality Index (PSQI) and bed/rise time of the residents. Logistic regression analysis and multiple linear regression analysis were used to examine the associations between sleep characteristics and MetS as well as MetS components. A generalized additive model was built to assess the smooth relationship between triglyceride (TG) levels and sleep duration.

**Results:**

Of the 1499 participants, 449 (30.0%) had MetS, and 443 (29.6%) had poor sleep quality. The rise time was found to be associated with MetS (> 6:00 vs. 5:00 ~ 6:00: adjusted OR (95%) = 1.77 (1.17–2.69), *P* = 0.007). For the MetS components, a U-shaped relationship was first revealed for sleep duration and TG levels (EDF = 1.85, *P* < 0.001). Furthermore, significant associations also included the associations of subjective sleep quality and daytime dysfunction with hypertension, the associations of sleep efficiency and rise time with hyperglycemia, the associations of rise time with TG levels, and the association of bedtime with waist circumference.

**Conclusions:**

The different sleep characteristics were associated with different MetS components.

**Supplementary Information:**

The online version contains supplementary material available at 10.1186/s12877-022-03074-8.

## Background

Metabolic syndrome (MetS) is a clustering of clinical syndromes with obesity, hyperglycemia (diabetes mellitus or impaired glucose regulation), dyslipidaemia (high serum triglyceride (TG) levels and/or low high-density lipoprotein cholesterol (HDL-C) levels) and hypertension. MetS is a common health problem among older adults, and its prevalence ranges from 24.5% to 30.5% among Chinese adults and ranges from 32.4% to 36.9% among older adults [1–3]. The pathogenic mechanisms of MetS are complex. Previous studies have shown that environmental factors and lifestyle factors play an important role [4]. Sleep as a modifiable lifestyle factor has been shown to be associated with MetS.

The prevalence of sleep disorder in China ranges from 47.2% to 49.9% for the population ≥ 60 years old [5, 6], and it often coexists with MetS in older population. Studies have shown that the Sleep curtailment affects human metabolism and endocrine function, and then affects the quality of life and health of older adults in the long term [7, 8]. Some studies have revealed the biological mechanism by which sleep contributes to the risk of MetS. Sleep disorders or short sleep duration can cause hypertension, overweight, obesity, type 2 diabetes, and dyslipidemia through affect sympathetic nerve activity [9], regulate leptin and ghrelin [10, 11], reduce insulin sensitivity, promote insulin resistance [12, 13], stimulate lipodieresis, increase plasma free fatty acid level [14]and other multiple biological mechanisms, all of which are risk factors for MetS. In the past few years, several studies have identified that sleep duration is associated with MetS [15–19] and MetS components such as waist circumference (WC), fasting plasma glucose (FPG) levels, TG levels, and HDL-C levels [20]. In addition, sleep characteristics based on Pittsburgh Sleep Quality Index (PSQI) were reported to be associated with MetS or MetS both in cross-sectional studies [21–23] and prospective study [24]. However, most studies only focused on individual sleep characteristic or global PSQI score. It is also worthwhile to observe which sleep characteristic has the best correlation with the specific MetS component. In addition, sleep characteristic profiles and their associations with MetS components among community-dwelling older adults have seldom been reported. Using a population-based cross-sectional study, the present study aimed to depict the sleep characteristics, including global PSQI quality, each of PSQI characteristics and rise/bed time, among older adults in China, and explore the correlation between these sleep characteristics and MetS components.

## Methods

### Participants and sampling

This population-based cross-sectional study was performed in Songjiang District, Shanghai, China, from May 1 2020 to December 31 2020. Participant recruitment was conducted using a multistage random sampling method. The samples of this study came from 15 blocks in Songjiang District. Neighborhoods with more than 200 residents aged ≥ 60 years were selected from 15 blocks. Among these selected neighborhoods, one neighborhood was randomly selected from each block and the list of home addresses was extracted. A total of 3534 permanent residents aged ≥ 60 years were selected and were invited to participate in the project. The neighborhood committee issued informed consent forms to these residents on the list. Informed consent forms stated that participants should have no diagnosed mental disorders and were physically able to conduct the survey. Participants came to the community health care center to participate in the investigation with signed informed consent forms, identity card and medical card. A total of 1646 participants aged ≥ 60 years came to participate in the investigation and none of the participants was found to have a medical record of mental disorders after a medical card was checked. Of the 1646 participants, 62 participants were missing sleep data, and 85 participants with a sleep efficiency greater than 1 which were considered as invalid data. Finally, 1499 participants were included in this study for analysis. Demographic information of the participants was collected via a face-to-face questionnaire survey, including age, sex, education level, marital status, depression, and lifestyles (drinking, smoking, sleep, physical activity, etc.).

This project was combined with the National Public Health Service Physical Examination Program for The Elderly. In the physical examination program, anthropometric indices, including WC, height and weight, were measured. Blood pressure (BP) was measured with a mercury sphygmomanometer. All participants provided venous blood samples after 12 h of overnight fasting. Fasting plasma glucose (FPG, hexokinase method), triglycerides (GPO-PAP), and high-density lipoprotein cholesterol (HDL-C, homogeneous enzyme immunoassay) were measured.

### Measures and definitions

#### Metabolic syndrome

Metabolic syndrome (MetS) was defined according to the guidelines for the prevention and treatment of dyslipidemia in Chinese adults (Revised Edition 2016). Participants who met at least three of the following conditions were defined as having MetS: (1) WC ≥ 90 cm in men, or ≥ 85 cm in women; (2) Hyperglycemia: FPG level ≥ 6.1 mmol/L or Oral Glucose Tolerance Test (OGTT) 2 h plasma glucose level ≥ 7.8 mmol/L, and/or previously diagnosed diabetes that was under treatment; (3) Hypertension: BP ≥ 140/90 mmHg and/or diagnosed with hypertension that was under treatment; (4) HDL-C level < 1.04 mmol/L; and (5) TG level ≥ 1.70 mmol/L.

#### Sleep characteristics

Sleep characteristics were assessed using the Pittsburgh Sleep Quality Index (PSQI) and bed/rise time. The PSQI is a 19-item self-report questionnaire that assesses sleep quality and disturbances during the last month and generates seven component scores: subjective sleep quality, sleep latency (time spent before falling asleep), sleep duration (nocturnal), sleep efficiency (proportion of sleep duration to total time spent in bed), sleep disturbances, sleep drug use, and daytime dysfunction. The sum of the scores for these seven components forms a global PSQI score that ranges from 0 to 21, and a higher PSQI score indicates worse sleep quality. A global PSQI score greater than 5 was defined to differentiate poor sleep quality from good sleep quality. Each of the 7 sleep characteristics was analyzed on a 0–3 scale. Subjective sleep quality (0-very good sleep quality; 1—general sleep quality; 2—bad sleep quality; 3—very bad sleep quality) and sleep latency (0—≤ 15 min, 1—16 ~ 30 min, 2—31 ~ 60 min, 3—> 60 min) were assessed from 0–3 points. Sleep efficiency was divided into three groups (0—≥ 85%; 1—75% ~ 84%; 2—< 75%). Sleep duration was divided into three groups (0—6–8 h; 1—< 6 h; 2—> 8 h). Sleep drugs were divided into two groups (score = 0 was defined as no use; scores = 1, 2 and 3 were defined as use). Sleep disturbances were divided into three groups (score = 0 was defined as no; score = 1 was defined as mild; scores = 2/3 were defined as moderate-severe). Daytime dysfunction was divided into two groups (score = 0 was defined as no; scores = 1, 2 and 3 were defined as yes). Rise time was classified into four groups: < 5:00, 5:00 ~ 6:00, 6:01 ~ 7:00, and > 7:00, and bed time was classified into four groups: < 21:00, 21:00 ~ 22:00, 22:01 ~ 23:00, and > 23:00 [25].

#### Covariates

Data on age, sex, marital status (married and single) and education levels (illiterate, primary school and ≥ junior school) were collected from participants' self-reports. BMI was calculated as weight in kilograms divided by height in metres squared. Smoking status was defined by two questions: “Have you smoked 100 cigarettes so far?” and “Have you smoked in the past 30 days?” According to the answers to these two questions, smoking was divided into three categories. Those who had not smoked 100 cigarettes so far were defined as never smokers; those who had smoked 100 cigarettes so far and had not smoked in the past 30 days were defined as people who given up smoking; those who had smoked 100 cigarettes so far and had smoked in the last 30 days were defined as current smokers. Drinking status was categorized as drinking and never drinking. Physical activity (PA) was assessed by two questions: “During the last week, how many days did you perform moderate-intensity physical activities, such as playing badminton, walking fast, playing table tennis and square dancing? (None, 1–2 times, 3–4 times, 5–6 times, 7 times or more)” and “How much time did you spend doing physical activities each time you performed them? (< 20 min, 20–30 min, 30-40 min, 40–50 min, > 50 min)” [26], and the product of these two answers was used to calculate the time interval of PA in a week. Based on the American Heart Association's recommendation [27] and current recommendations for the practicing of physical activity [28], PA was categorized as inactive (PA < 150 min/week) and active (PA ≥ 150 min/week). Depression was assessed using the Patient Health Questionnaire-9 (PHQ-9), which consists of 9 questions. The scores for each question range from 0 (not at all) to 1 (several days), 2 (more than half of the days), and 3 (nearly every day). The total score can range from 0–27 points. The participants were divided into five groups based on the total score (0–4: no depression, 5–9: mild depression, 10–14: moderate depression, 15–19: moderate to severe depression, and 20–27: major depression).

### Statistical analysis

Sociodemographic and sleep characteristics of the participants were shown using descriptive statistical methods. Comparisons were performed using t tests for continuous variables between the MetS group and the non-MetS group and chi-square tests for categorical variables. Odds ratios (ORs) and 95% confidence intervals (CIs) were estimated using logistic regression model for the analysis of MetS’s association with the global sleep quality (poor sleep quality: PSQI score > 5, good sleep quality: PSQI score ≤ 5) and each PSQI characteristic. The associations of MetS components with global sleep quality or with each of 7 PSQI characteristics were furtherly analyzed. Multiple linear regression models were used for association between three continuous variables components of MetS (WC levels, TG levels and HDL-C levels) and sleep characteristics and multivariate logistic regression analysis were used for the association between hyperglycemia and hypertension (as a dichotomous variable according to the diagnostic criteria of metabolic syndrome) and sleep characteristics. Adjustments for age, sex, BMI, drinking status, smoking status, PA, and depression were made in all the multivariate analyses mentioned above. To examine nonlinear relationships between sleep duration and MetS/each MetS components, a generalized additive model (GAM) was built to explore their smooth relationships. Age, sex, BMI, smoking status, drinking status, PA and depression were applied to adjust the GAM. All analyses were performed using STATA software (version 16.0) and R (version 3.6.3), and a *P* < 0.05 was defined as statistically significant.

## Results

### Participants characteristics

The characteristics of participant are displayed in Table 1. The mean age of all 1499 participants was 72.39 ± 5.65 years (range 65–101 years), and the mean BMI was 24.08 ± 3.54 kg/m^2^. The majority of the participants were female (55.4%), illiterate (44.3%), married (82.4%), never drinkers (83.2%), never smokers (75.9%), physically inactive (55.8%), and not depressed (86.6%).

**Table 1:** Socio-demographic and sleep characteristics of adults by metabolic syndrome

Variable	Total (*n* = 1499)	Metabolic syndrome		*P*
		**Yes (*n* = 449)**	**No (*n* = 1050)**	
Age	72.39 ± 5.65	71.81 ± 5.41	72.64 ± 5.74	0.008
Gender				< 0.001
male	668(44.6)	161(35.9)	507(48.3)	
female	831(55.4)	288(64.1)	543(51.7)	
BMI	24.08 ± 3.54	26.32 ± 3.26	23.12 ± 3.20	< 0.001
Educational attainment				0.644
Illiteracy	664(44.3)	207(46.1)	457(43.5)	
Primary school	549(36.6)	158(35.2)	391(37.2)	
junior school	286(19.1)	84(18.7)	202(19.2)	
Marital				0.053
Single	264(17.6)	66(14.7)	198(18.9)	
Married	1235(82.4)	383(85.3)	852(81.8)	
Smoking				0.974
Never	1137(75.9)	342(76.2)	795(75.7)	
Former	93(6.2)	27(6.0)	66(6.3)	
Current	269(17.9)	80(17.4)	189(18.0)	
Alcohol use				0.199
Never	1247(83.2)	365(81.3)	882(84.0)	
Drinker	252(16.8)	84(18.7)	168(16.0)	
Physical activity				0.346
Active	662(44.2)	259(57.7)	578(55.0)	
Inactive	837(55.8)	190(42.3)	472(45.0)	
Depression				0.357
No depression	1298(86.6)	390(86.9)	908(86.5)	
Mild depression	151(10.1)	40(8.9)	111(10.6)	
Moderate to depression	27(1.8)	8(1.8)	19(1.8)	
Moderate to severe depression	19(1.3)	9(2.0)	10(1.0)	
Major depression	4(0.3)	2(0.4)	2(0.2)	
PSQI				
Subjective sleep quality				0.463
Very good	490(32.7)	153(34.1)	337(32.1)	
General	791(52.8)	229(51.0)	562(53.5)	
Bad	186(12.4)	54(12.0)	132(12.6)	
Very bad	32(2.1)	13(2.9)	19(1.8)	
Sleep efficiency				0.172
≥ 85%	843(56.2)	254(56.6)	589(56.1)	
75% ~ 84%	303(20.2)	101(22.5)	202(19.2)	
< 75%	353(23.5)	94(20.9)	159(24.7)	
Sleep duration				0.824
6-8 h	1134(75.7)	336(74.8)	798(76.0)	
< 6 h	180(12.0)	54(12.0)	126(12.0)	
> 8 h	185(12.3)	59(13.1)	126(12.0)	
Sleep latency				0.111
≤ 15 min	540(36.0)	161(35.9)	379(36.1)	
16-30 min	654(43.6)	196(43.7)	458(43.6)	
31-60 min	165(11.0)	40(8.9)	125(11.9)	
> 60 min	140(9.3)	52(11.6)	88(8.4)	
Sleep drug				0.821
Not use	1412(94.2)	422(94.0)	990(94.3)	
Use	87(5.8)	27(6.0)	60(5.7)	
Sleep disturbances				0.180
None	360(24.0)	118(26.3)	242(23.0)	
Mild	1051(70.1)	306(68.2)	745(71.0)	
Moderate-severe	88(5.9)	25(5.6)	63(6.0)	
Daytime dysfunction				0.438
None	980(65.4)	287(63.9)	693(66.0)	
Yes	519(34.6)	162(36.1)	357(34.0)	
PSQI score	4.69 ± 3.28	4.71 ± 3.47	4.69 ± 3.20	0.915
PSQI				0.482
PSQI ≤ 5	1056(70.4)	322(71.7)	734(69.9)	
PSQI > 5	443(29.6)	127(28.3)	316(30.1)	
Bed time				0.592
< 21:00	599(40.0)	183(40.8)	416(39.6)	
21:00 ~ 22:00	849(56.6)	248(55.2)	601(57.2)	
22:01 ~ 23:00	42(2.8)	16(3.6)	26(2.5)	
> 23:00	9(0.6)	2(0.4)	7(0.7)	
Rise time				0.199
< 5:00	221(14.7)	61(13.6)	160(15.2)	
5:00 ~ 6:00	1146(76.5)	338(75.3)	808(77.0)	
6:01 ~ 7:00	116(7.7)	44(9.8)	72(6.9)	
> 7:00	16(1.1)	6(1.3)	10(0.9)	

*BMI* Body Mass Index

*PSQI* the Pittsburgh Sleep Quality Index

Of all the participants, 29.6% had poor sleep quality (PSQI score > 5), 14.5% had bad/very bad subjective sleep quality, 76.0% had mild/moderate-severe sleep disturbances and 34.6% had daytime dysfunction. The majority of older adults had normal sleep duration (6-8 h) (75.7%), went to bed at 21:00 ~ 22:00 PM (56.6%) and got up at 5:00 ~ 6:00 AM (76.5%). Of all participants, 30.0% had MetS. Participants with MetS were slightly, but statistically significantly younger (71.81 vs. 72.64, *P* = 0.008), and had higher BMI level (26.32 vs. 23.12, *P* < 0.001) and higher prevalence of female (64.1% vs. 51.7%, *P* < 0.001) than those without MetS. There were no differences in the distribution of global PSQI scores, any of PSQI characteristic, bed time and rise time between MetS group and non-MetS group.

### Relationship between sleep characteristics and MetS

The results of logistic regression analysis on the associations between sleep characteristics and MetS after adjusting for age, sex, BMI, smoking status, drinking status, PA and depression are shown in Table 2. Older adults with rise time > 6:00 had higher odds of being MetS compared to those with rise time of 5:00 ~ 6:00 (univariate analysis: OR = 1.46, 95% CI 1.00–2.12, *P* = 0.048; multivariate analysis: OR = 1.77, 95% CI 1.17–2.69, *P* = 0.007). The global PSQI score and each PSQI characteristic had no significant association with MetS.

**Table 2:** Odds ratios (with 95% CIs) of metabolic syndrome on the basis of sleep characteristics

	**Model 1**		**Model 2**	
	**OR (95%CI)**^**a**^	***P***	**OR (95%CI)**^**b**^	***P***
PSQI				
PSQI ≤ 5	Reference		Reference	
PSQI > 5	0.92(0.72,1.17)	0.482	0.87(0.66,1.16)	0.341
Subjective sleep quality				
Very good	Reference		Reference	
General	0.89(0.70,1.15)	0.387	0.90(0.68,1.19)	0.443
Bad	0.90(0.62,1.30)	0.581	0.85(0.56,1.31)	0.471
Very bad	1.51(0.73,3.13)	0.271	1.05(0.46,2.41)	0.905
Sleep efficiency				
≥ 85%	Reference		Reference	
75% ~ 84%	1.16(0.88,1.53)	0.301	1.24(0.90,1.70)	0.191
< 75%	0.84(0.64,1.11)	0.224	0.91(0.66,1.26)	0.575
Sleep latency				
≤ 15 min	Reference		Reference	
16-30 min	1.01(0.79,1.29)	0.954	1.05(0.79,1.39	0.743
31-60 min	0.75(0.50,1.12)	0.166	0.72(0.46,1.14)	0.164
> 60 min	1.39(0.94,2.05)	0.097	1.32(0.84,2.08)	0.225
Sleep duration				
6-8 h	Reference		Reference	
< 6 h	1.02(0.72,1.43)	0.92	0.98(0.66,1.45)	0.927
> 8 h	1.11(0.80,1.55)	0.533	1.31(0.90,1.91)	0.155
Sleep disturbances				
None	Reference		Reference	
Mild	0.84(0.65,1.09)	0.191	0.85(0.63,1.13)	0.261
Moderate-severe	0.81(0.49,1.36)	0.180	0.61(0.34,1.12)	0.112
Sleep drug				
Not use	Reference		Reference	
Use	1.06(0.66,1.69)	0.821	1.04(0.61,1.78)	0.888
Daytime dysfunction				
None	Reference		Reference	
Yes	1.10(0.87,1.38)	0.438	1.01(0.77,1.33)	0.951
Bed time				
21:00 ~ 22:00	Reference		Reference	
< 21:00	1.07(0.85,1.34)	0.583	1.16(0.88,1.51)	0.289
> 22:00	1.32(0.73,2.39)	0.356	1.05(0.54,2.05)	0.893
Rise time				
5:00 ~ 6:00	Reference		Reference	
< 5:00	0.91(0.66,1.26)	0.571	0.86(0.60,1.24)	0.421
> 6:00	1.46(1.00,2.12)	0.048	1.77(1.17,2.69)	0.007

*PSQI* the Pittsburgh Sleep Quality Index

*OR* Odds ratios

*CI* confidence interval

^a^Unadjusted

^b^Adjusted for age, sex, BMI, smoking status, alcohol use status, physical activity and depression

### Sleep characteristic profiles and MetS spectrum

**Fig. 1:**
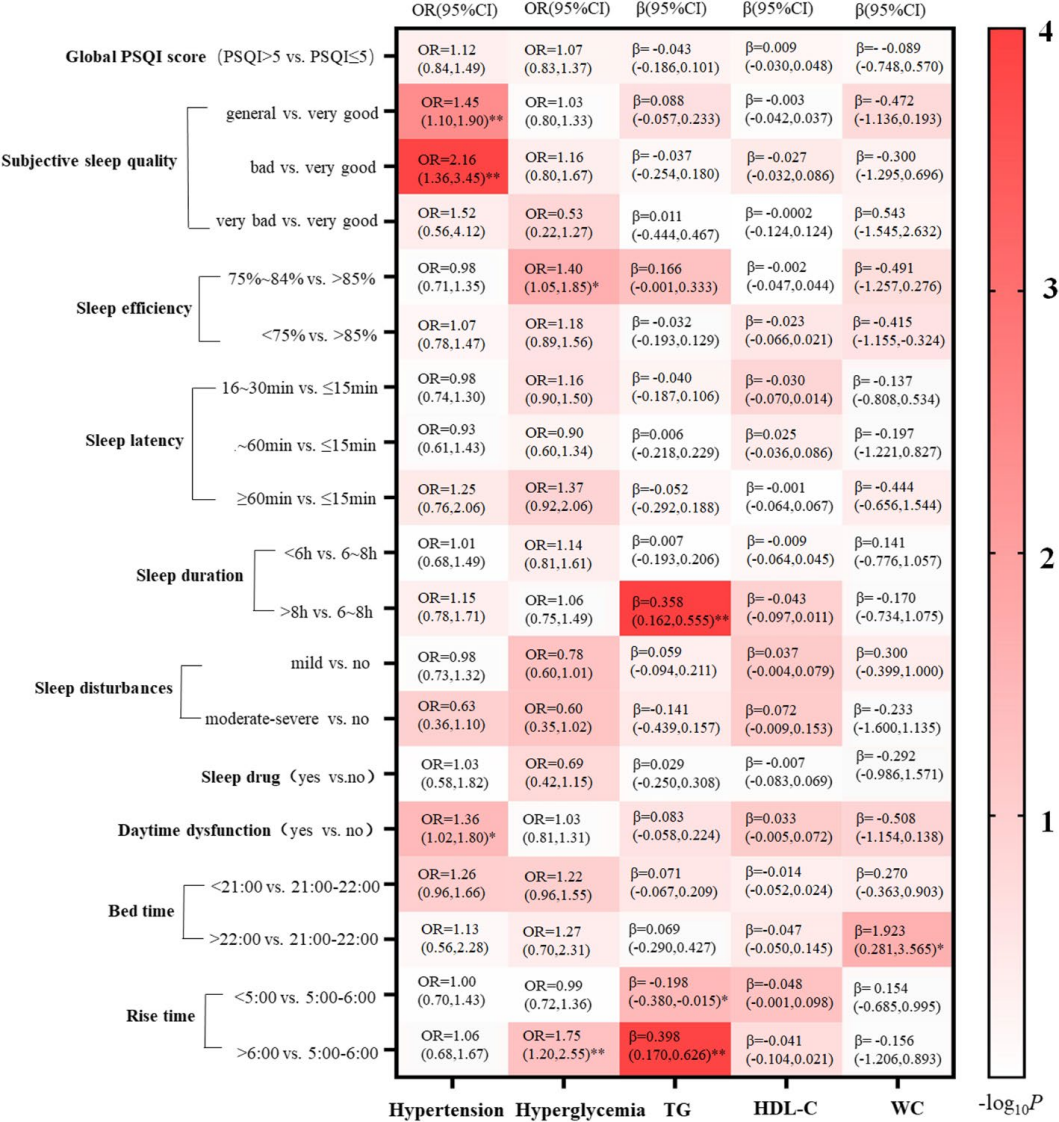
Relationship between sleep characteristics and MetS components. Odds ratios (ORs) and 95% confidence intervals (CIs) were estimated using logistic regression model for sleep characteristics’ association with hypertension and hyperglycemia. The standardized regression coefficients (β) and 95% CI was estimated using multiple linear regression model for sleep characteristics’ association with WC levels, TG levels and HDL-C levels. The OR and β were adjusted for age, sex, BMI, smoking status, alcohol use status, physical activity and depression. P values were presented with asterisks: *P < 0.05, **P < 0.01

**Fig. 2:**
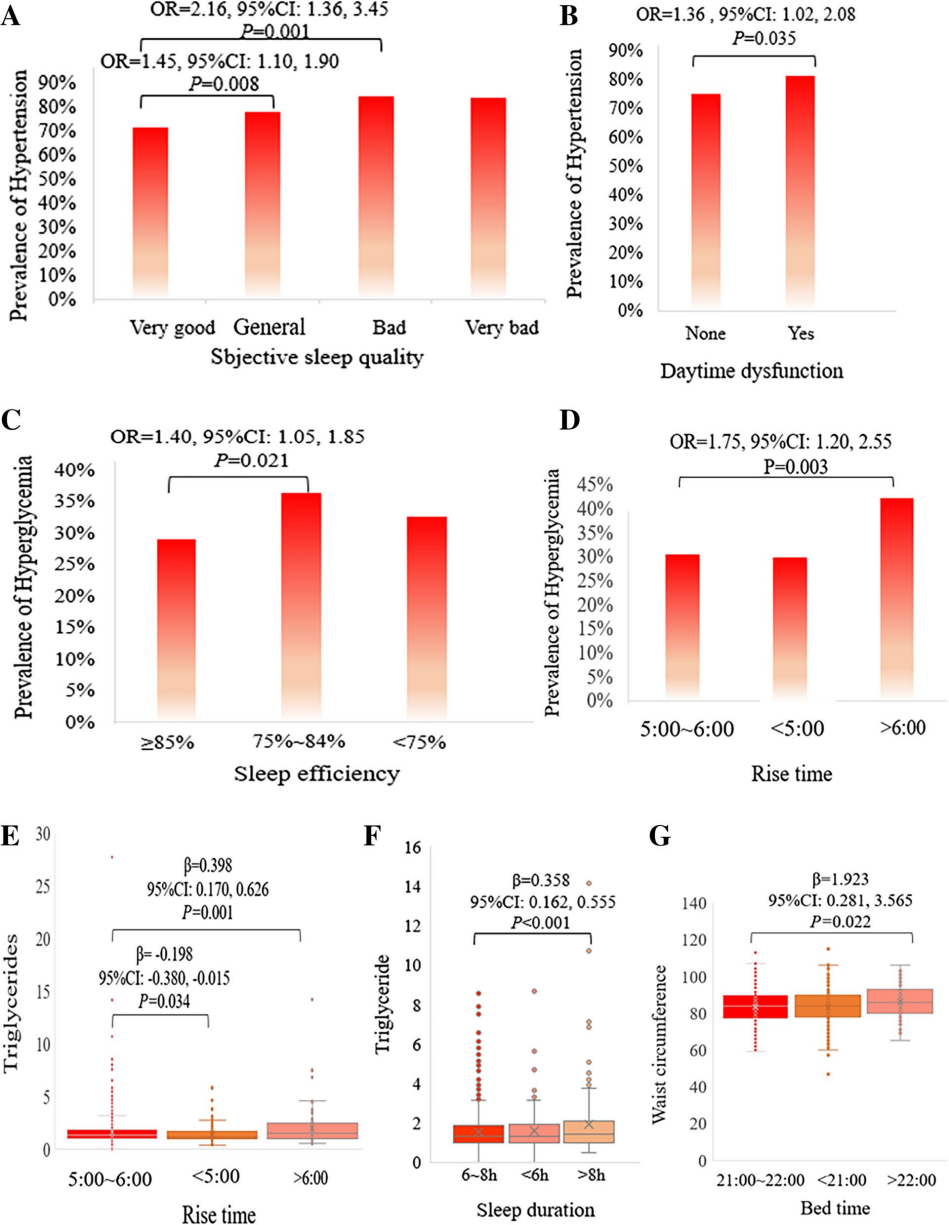
Specific description of significant associations between sleep characteristics and MetS components. Subjective sleep quality **(A)** and Daytime dysfunction **(B)** was associated with hypertension. Sleep efficiency **(C)** and rise time **(D)** was associated with hyperglycemia. Rise time **(E)** and sleep duration **(F)** was associated with triglyceride levels. **(G)** Bed time was associated with WC levels

Figure 1 shows the significant associations between each PSQI characteristic and MetS component, including of significant associations of subjective sleep quality and daytime dysfunction with hypertension, the associations of sleep efficiency and rise time with hyperglycemia, the associations of sleep duration and rise time with TG levels, and the association of bed time with WC. Specifically, participants with general quality (OR = 1.45, 95% CI 1.10–1.90, *P* = 0.008) and bad subjective sleep quality (OR = 2.16, 95% CI 1.36–3.45, *P* = 0.001) had higher odds of being hypertension compared with those with very good subjective sleep quality (Fig. 2A). Participants undergoing daytime dysfunction have higher odds of being hypertension compared with those without daytime dysfunction. (OR = 1.36, 95% CI 1.02–1.80, *P* = 0.035, Fig. 2B). For the hyperglycemia, the chance of having an association with hyperglycemia was 1.40 times higher for the participants with a sleep efficiency of 75% ~ 84% than those with a sleep efficiency of ≥ 85% (OR = 1.40, 95% CI 1.05–1.85, *P* = 0.021, Fig. 2C), and participants with rise time > 6:00 also had higher odds of being hyperglycemia than those with rise time 5:00 ~ 6:00 (OR = 1.75, 95%CI 1.20–2.55, *P* = 0.003, Fig. 2D). With respect to the TG, participants with rise time > 6:00 had higher TG levels (β = 0.398, 95% 0.170–0.626, *P* = 0.001, Fig. 2E) than those with rise time of 5:00 ~ 6:00, and participants with rise time < 5:00 had lower TG levels (β = -0.198, 95% -0.380- -0.015, *P* = 0.034, Fig. 2E) than those with rise time of 5:00 ~ 6:00. In addition, participants with sleep duration of more than > 8 h had higher TG levels than those with sleep duration of 6 ~ 8 h hours (β = 0.358, 95% 0.162–0.555, *P* < 0.001, Fig. 2F). Moreover, participants with bed time > 22:00 have higher WC levels compared to those with bed time 21:00 ~ 22:00 (β = 1.923, 95% 0.281–3.565, *P* = 0.022, Fig. 2G).

**Fig. 3:**
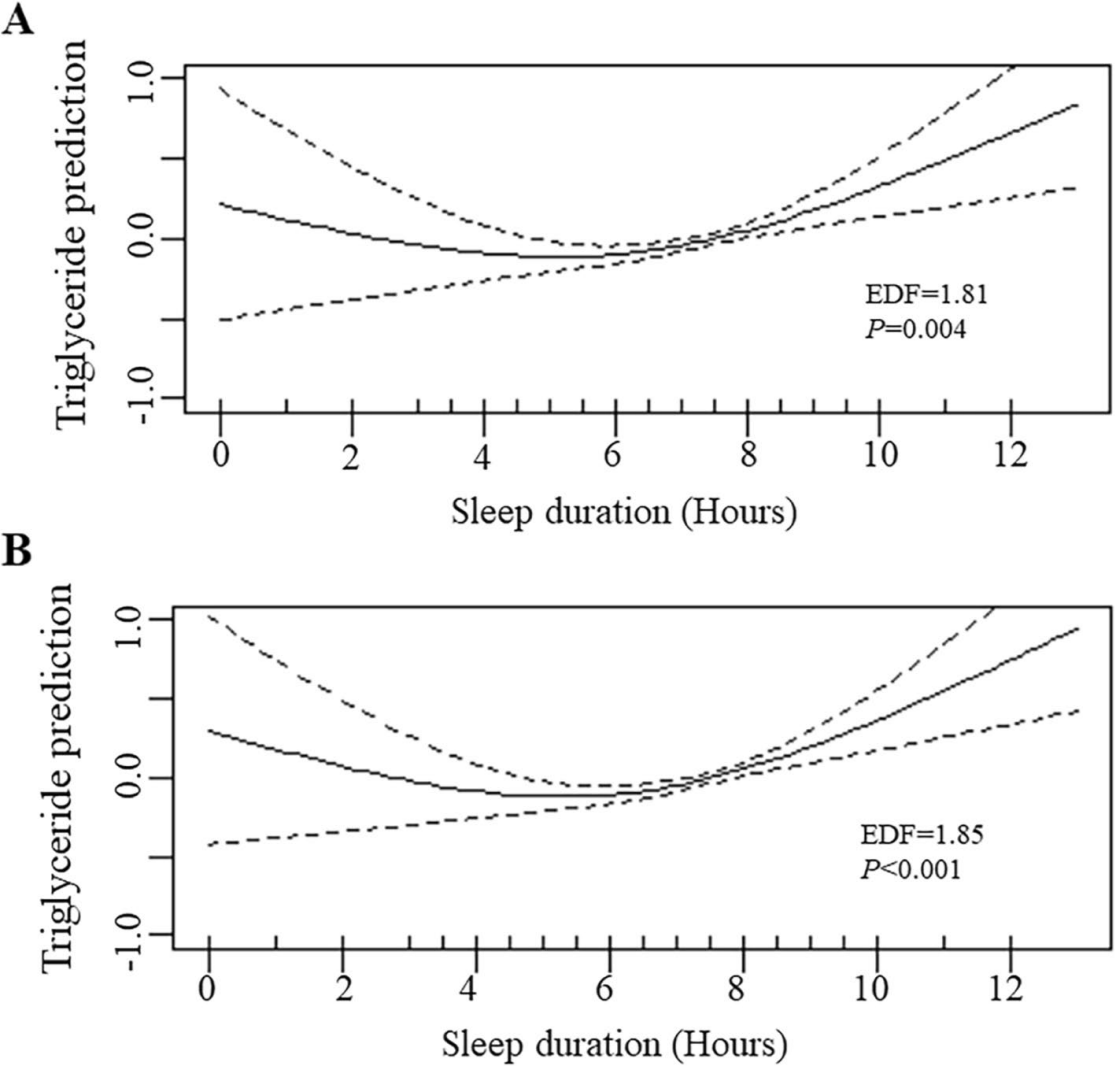
Plots of estimated smoothing spline function of sleep duration with 95% confidence intervals for the generalized additive model when the response variable was triglyceride levels. **(A)** Model 1 shows the univariable smooth function of sleep duration (EDF = 1.81, *P* = 0.004). **(B)** Model 2 shows the multivariable smooth function of sleep duration, adjusted for age, sex, BMI, smoking status, alcohol use status, physical activity and depression (EDF = 1.85, *P* < 0.001)

The nonlinear relationship between sleep duration and TG levels was further probed by the generalized additive model. As shown in Fig. 3, a U-shaped association with a significant nonlinear fit between sleep duration and TG levels was found both in the univariable GAM (EDF = 1.81, *P* = 0.004) and in the multivariable GAM. (EDF = 1.85, *P* < 0.001).

## Discussion

The present study demonstrated that several sleep characteristics were associated with metabolic syndrome (MetS) or the component of MetS in older Chinese adults, including poor subjective sleep quality, sleep insufficiency, daytime dysfunction, a sleep duration that was too long or too short, go to bed late and waking up late.

We first reported that older adults with rise time > 6:00 had higher odds of being MetS and hyperglycemia, and higher triglyceride (TG) levels, compared to those with rise time 5:00 ~ 6:00. In addition, we also found that older adults with bed time > 22:00 had higher waist circumference (WC) levels than those with bed time 21:00 ~ 22:00. These results indicated that sleep–wake cycle might influence the risk of MetS through regulating the glucose, WC and TG levels. We are rarely aware of any published study that has examined the association between the rise/bed time and MetS components. To our knowledge, only one study conducted among Iranian adults reported that waking earlier than 7:00 am was associated with MetS compared to waking later than 7:00 am in univariate analysis, but this association was not observed in multivariate analysis [22]. The sleep–wake cycle was reported to have a markedly modulatory effect on glucose tolerance and insulin secretion [29]. The sleep–wake cycle was also found to be linked to various processes of metabolism and sleep by affecting the circadian system [30]. Additionally, in our study, 96.6% older adults have bed time ≤ 22:00, indicating that waking up late means a longer sleep duration, and the association between long sleep duration and MetS and its components has been demonstrated [31].

Our results showed a strong U-shaped association between sleep duration and TG levels in older adults. A positive associations were previously reported between a long sleep duration and elevated TG levels [18, 20], as well as between a short sleep duration and elevated TG levels [32]. In addition, a U-shaped association between sleep duration and MetS was observed in a cross-sectional study among America populations [15], and both short sleep durations [33–35] and long sleep durations [16, 18, 36] were found to be associated with MetS [37]. Despite the study type, diagnostic criteria, risk classification, and sample characteristics of these studies, the association between sleep duration and MetS has been consistent. High TG levels has been confirmed as a risk factor for MetS [38], therefore, our findings indicated that sleep duration might influence the chance of being MetS partially through influencing the TG levels.

In our study, worse subjective sleep quality and daytime dysfunction were found to be associated with hypertension. A consistent positive association between poor subjective sleep quality and hypertension were previously reported in Chinese adults [39] and in Italian populations [40]. Several studies have suggested that poor sleep quality was associated with higher levels of diastolic blood pressure and/or systolic blood pressure [41–43]. For the association between daytime dysfunction and hypertension in older adults, the consistent result was also observed in Chinese adults [39]. However, the biological mechanisms of the association between these sleep characteristics and hypertension have not been elucidated. It has been reported that sleep may affect blood pressure by affecting sympathetic nerve activity to the vasculature and that abnormal sleeping may be involved in the pathogenesis of nondipping prehypertension and subsequently in hypertension disturbances [44].

We also found that sleep inefficiency was associated with hyperglycemia. In our study, hyperglycemia consists of fasting plasma glucose (FPG level) ≥ 6.1 mmol/L or Oral Glucose Tolerance Test (OGTT) 2 h plasma glucose level ≥ 7.8 mmol/L and/or previously diagnosed diabetes that was under treatment. In line with the result, a community-based cross-sectional study showed an inverse association between poor sleep efficiency and prevalence of diabetes in middle-aged and older adults [45]. Additionally, a study found that poor sleep efficiency was significantly associated with worse glycemic control among type 2 diabetic patients [46]. Some previously findings can provide the possible mechanism underlying the association between sleep inefficiency and hyperglycemia. Sleep inefficiency is a common quantitative indicator to evaluate insomnia [45, 47], and individuals with poor sleep efficiency are more likely to have poor sleep quality, which affects glucose regulation by changing levels of leptin [48, 49]. In addition, sleep inefficiency may induce diabetes risk factors by association with hypothalamic–pituitary–adrenal axis activity [50, 51]. Moreover, studies have indicated that poor sleep efficiency may cause reduced insulin sensitivity, impaired glucose tolerance, and decreased acute insulin resistance [7, 48].

For the analyses on association between the sleep efficiency and MetS as well as MetS components, we discard participants that have sleep efficiency > 1 due to possible self-reported error or participants' carelessness. Considering that their sleep may also be efficient, we included the participants whose sleep efficiency greater than 1 and classified them into group of sleep efficiency ≥ 85%. We also found that participants with a sleep efficiency of 75% ~ 84% have higher odds of being hyperglycemia compared to those with a sleep efficiency of ≥ 85% (OR = 1.39, 95% CI 1.05–1.89, *P* = 0.023, Supplementary Table S1).

Several limitations in this study should be acknowledged. First, this study is a cross-sectional study, and the ability to address the causal relationship between sleep and MetS is restricted. Therefore, this result should be further confirmed in a prospective cohort study. Second, this study only assessed older Chinese adults living in Shanghai, thus potentially limiting the generalizability of these results. Third, we evaluated sleep characteristics by self-report questionnaires, which may result in information bias. In addition, obstructive sleep apnoea, which can lead to poor sleep quality and sleep fragmentation, was not considered in this study. However, the PSQI scale includes snoring, which is a major symptom of obstructive sleep apnoea and can be considered as a risk indicator of sleep apnoea. Finally, in this survey-based study in a community population, we did not obtain accurate medication information for the older adults. However, the definition of the component of MetS includes the laboratory parameters and the medical histories of disease, such as diabetes and hypertension, which may reduce, to some extent, the impact of non-medication information on the results.

## Conclusion

In summary, the rise time was an important sleep characteristic that may influence MetS for older adults. Poor subjective sleep quality, sleep insufficiency, daytime dysfunction, sleep duration that were too long or too short, and rise late were associated with different MetS components, here evidenced for adults aged 60 y and older. These findings indicated that different sleep characteristics were associate MetS through different pathways, and future longitudinal studies are necessary to confirm these results.

## Supplementary Information

**Additional file 1:** **Table S1.**The association between sleep efficiency and MetS and MetS compinents. (Included the participants whosesleep efficiency greater than 1 and classified them into group of sleep efficiency≥ 85%.).

## Data Availability

The data that support the findings of this study are available from The Songjiang Center of Disease Prevention and Control, but restrictions apply to the availability of these data, which were used under license for the current study, and so are not publicly available. Data are however available from the corresponding author Qianyi Xiao upon reasonable request and with permission of The Songjiang Center of Disease Prevention and Control.

## Abbreviations

PSQI: Pittsburgh Sleep Quality Index
MetS: Metabolic Syndrome
BP: Blood pressure
HDL-C: High-density lipoprotein cholesterol
TG: Triglyceride
WC: Waist circumference
FPG: Fasting plasma glucose
PA: Physical Activity
ORs: Odds ratios
CI: Confidence Intervals
BMI: Body Mass Index
GAM: Generalized additive model
