# Supplementary material for: Sleep characteristic profiles and the correlation with spectrum of metabolic syndrome among older adult: a cross-sectional study
Source: BMC Geriatr. 2022 May 11;22:414. doi: 10.1186/s12877-022-03074-8 (PMC9097235; doi:10.1186/s12877-022-03074-8)
Supplement: Supplementary file 1 — Additional file 1: Table S1.The association between sleep efficiency and MetS and MetS compinents. (Included the participants whosesleep efficiency greater than 1 and classified them into group of sleep efficiency≥ 85%.). [file 12877_2022_3074_MOESM1_ESM.docx]

|  | Mets | | hypertension | | hyperglycemia | | WC | | TG | | HDL | |
| --- | --- | --- | --- | --- | --- | --- | --- | --- | --- | --- | --- | --- |
|  | OR(95%CI) | P | OR(95%CI) | P | OR(95%CI) | P | OR(95%CI) | P | OR(95%CI) | P | OR(95%CI) | P |
| Sleep efficiency |  |  |  |  |  |  |  |  |  |  |  |  |
| ≥ 85% | ref |  | ref |  | ref |  | ref |  | ref |  | ref |  |
| 75%~ 84% | 1.24  (0.90,1.71) | 0.181 | 0.97  (0.71,1.35) | 0.875 | 1.39  (1.05,1.85) | 0.023 | -0.49  (-1.26,0.28) | 0.210 | 0.17  (-0.001,0.33) | 0.051 | -0.002  (-0.05,0.05) | 0.927 |
| <75% | 0.86  (0.64,1.16) | 0.315 | 0.87  (0.66,1.15) | 0.332 | 1.10  (0.85,1.43) | 0.478 | -0.40  (-1.14,0.33) | 0.284 | -0.03  (-0.19,0.13) | 0.692 | -0.02  (-0.07,0.02) | 0.281 |

**Supplementary Table S1:**The association between sleep efficiency and MetS and MetS compinents. (Included the participants whose sleep efficiency greater than 1 and classified them into group of sleep efficiency ≥ 85%.)
